# Identification of compounds responsible for the anthelmintic effects of chicory (*Cichorium intybus*) by molecular networking and bio-guided fractionation

**DOI:** 10.1016/j.ijpddr.2021.02.002

**Published:** 2021-02-11

**Authors:** Angela H. Valente, Matthew de Roode, Madeleine Ernst, Miguel Peña-Espinoza, Louis Bornancin, Charlotte S. Bonde, María Martínez-Valladares, Sabrina Ramünke, Jürgen Krücken, Henrik T. Simonsen, Stig M. Thamsborg, Andrew R. Williams

**Affiliations:** a Department of Veterinary and Animal Sciences, Faculty of Health and Medical Sciences, University of Copenhagen, Frederiksberg, Denmark; b Sensus b.v., Oostelijke Havendijk 15, 4704, RA Roosendaal, the Netherlands; c Section for Clinical Mass Spectrometry, Danish Center for Neonatal Screening, Department of Congenital Disorders, Statens Serum Institut, Copenhagen, Denmark; d Instituto de Farmacología y Morfofisiología, Facultad de Ciencias Veterinarias, Universidad Austral de Chile, Valdivia, Chile; e Department of Biotechnology and Biomedicine, Technical University of Denmark, Søltofts Plads 223, 2800, Kongens Lyngby, Denmark; f Instituto de Ganadería de Montaña, CSIC-Universidad de León, Department of Animal Health, 24346, Grulleros. León, Spain; g Institute for Parasitology and Tropical Veterinary Medicine, Freie Universität Berlin, Robert-von-Ostertag-Str. 7-13, 14163, Berlin, Germany

**Keywords:** Chicory, Anthelmintic, Parasite, Sesquiterpene lactones

## Abstract

Increasing resistance towards anthelmintic drugs has necessitated the search for alternative treatments for the control of gastrointestinal nematode parasites. Animals fed on chicory (*Cichorium intybus* L.), a temperate (pasture) crop, have reduced parasite burdens, hence making *C. intybus* a potentially useful source for novel anthelmintic compounds or a diet-based preventive/therapeutic option. Here, we utilized *in vitro* bioassays with the parasitic nematode *Ascaris suum* and molecular networking techniques with five chicory cultivars to identify putative active compounds. Network analysis predicted sesquiterpene lactones (SL) as the most likely group of anthelmintic compounds. Further bioassay-guided fractionation supported these predictions, and isolation of pure compounds demonstrated that the SL 8-deoxylactucin (8-DOL) is the compound most strongly associated with anti-parasitic activity. Furthermore, we showed that 8-DOL acts in a synergistic combination with other SL to exert the anti-parasitic effects. Finally, we established that chicory-derived extracts also showed activity against two ruminant nematodes (*Teladorsagia circumcincta* and *Cooperia oncophora*) in *in vitro* assays. Collectively, our results confirm the anti-parasitic activity of chicory against a range of nematodes, and pave the way for targeted extraction of active compounds or selective breeding of specific cultivars to optimize its future use in human and veterinary medicine.

## Highlights

• Molecular networking from extracts of multiple cultivars of chicory predicted anthelmintic compounds.
• Fractionation and isolation revealed 8-deoxylactucin (8-DOL) to be primarily responsible for anti-parasitic activity.
• 8-DOL acts synergistically with other sesquiterpene lactones to exert activity.

## Graphical abstract

**Figure:**
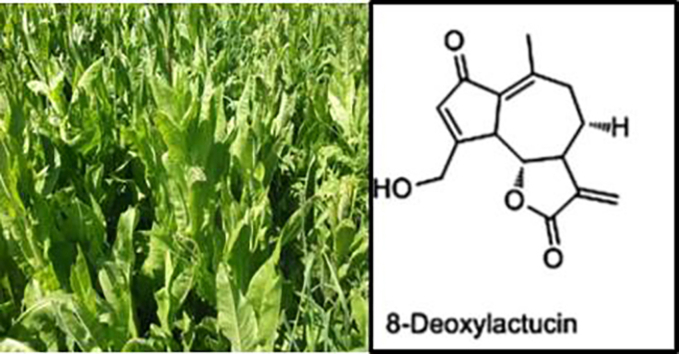

## 1 Introduction

Parasitic infections have major detrimental impacts on human populations worldwide, causing disease, reduced nutrition and quality of life, and reduced productivity of affected individuals. On a global scale, approximately 900 million people are infected with nematode helminths, mainly in tropical regions (Sangster et al., 2018). Livestock are also markedly affected by helminth parasites, which cause both clinical disease as well as subclinical effects on welfare and productivity (Charlier et al., 2014). Due to the absence of vaccines for most helminth infections, control of both human and livestock helminths relies almost entirely on effective anthelmintic drugs. However, parasite resistance to existing drugs is widespread and new treatment options are urgently required (Krücken et al., 2017; Sangster et al., 2018; Morgan et al., 2019).

Plants are a rich source of novel anti-parasitic compounds*.* This has been especially well demonstrated in livestock, where either wild plants or crops can be used as dietary interventions that may reduce intestinal helminth infection (Hoste et al., 2012; Pena-Espinoza et al., 2016). Whilst several laboratory studies have been performed on the use of new, plant-based treatment options against helminths, only a few have proved to be effective in *in vivo* trials (Bahuaud et al., 2006; Athanasiadou et al., 2007; Novobilský et al., 2013; Williams et al., 2014; Ropiak et al., 2016; Karonen et al., 2020). One of the most promising plant-based treatment options is chicory (*Cichorium intybus* L.), a perennial plant from the Asteraceae family. Chicory leaves are highly nutritious and can be used as both a forage for grazing livestock or for human salad consumption, whilst the roots are an abundant source of the polysaccharide inulin, extensively used as a feed additive and prebiotic. There is consistent evidence demonstrating anthelmintic effects in livestock fed chicory leaves over the last two decades (Peña-Espinoza et al., 2018). Cattle fed chicory-rich diets, either fresh by grazing or ensiled, have significantly reduced infection levels with the stomach nematode *Ostertagia ostertagi*, as compared to cattle offered ryegrass/clover mixes (Pena-Espinoza et al., 2016). Similarly, chicory feeding was found to reduce nematode infection levels in the stomach of lambs (Marley et al., 2003). Thus, chicory may be a natural source for a potential new generation of anthelmintic compounds, and/or used as a nutraceutical to control infections.

Anthelmintic effects from plants are thought to derive from bioactive compounds such as alkaloids, polyphenols, and terpenoids (Cowan, 1999). Chicory contains a wide range of phytochemicals with proposed bioactivity against several pathologies and infections. The four main groups are hydroxycinnamic acids, coumarins, flavonoids and sesquiterpene lactones (SL) (Rees and Harborne, 1985; Ferioli and D'Antuono, 2012). Several studies have suggested, based on *in vitro* experiments, that SL could be responsible for the anti-parasitic activity of chicory (Peña-Espinoza et al., 2018). The known SL in chicory are lactucin (LAC), 8-deoxylactucin (8-DOL), lactucopicrin (LACP), 11,13-dihydro-LAC, 11,13-dihydro-8-DOL and 11,13-dihydro-LACP. A positive correlation has been shown between increasing concentrations of 8-DOL in chicory extracts and the inhibition of egg hatching in free-living stages of the pathogenic nematode *Haemonchus contortus* (Foster et al., 2011). Subsequently, further *in vitro* assays with *O. ostertagi* and the swine helminths, *Oesophagostomum dentatum* and *Ascaris suum* (closely related to the human *A. lumbricoides*) also found marked differences in anti-parasitic effects between chicory cultivars, which correlated with distinct SL profiles (Peña-Espinoza et al., 2015; Williams et al., 2016). More recently, the activity of semi-purified extracts was investigated against *Caenorhabditis elegans* and *A. suum* and combined with *in silico* analysis in order to calculate global bioactivity scores on all compounds present in the extracts (Peña-Espinoza et al., 2020). SL were found to be the only compounds with significant bioactivity scores, suggestive of a role in the observed anthelmintic effects. SL are bioactive terpenoids commonly found in Asteraceae plants and mainly function by the lactone ring and its α,β- or α,β,χ−unsaturated carbonyl structures that react with thiol groups of cysteine residues in proteins, thus their biological activity is well established (Drew et al., 2009).

Whilst the active group of compounds has been proposed to be SL (Molan et al., 2003), definitive evidence of this is lacking. Here, we used a combination of bioactivity-based molecular networking and bio-guided fractionation to elucidate potential active compounds in chicory. Using material from multiple chicory cultivars, we utilized a robust anti-parasitic assay with *A. suum* larvae to assess the activity of extracts and fractions as well as purified compounds. This approach allowed us to definitively identify SL as the major anti-parasitic compounds, and address the question of whether this activity is derived from a single entity or a synergistic action of several distinct compounds. In addition, we conducted bioassays with parasitic nematodes from different hosts to establish that extracts from chicory exert broad-spectrum anti-parasitic activity.

## 2 Materials and methods

### 2.1 Plant materials

Root and leaf material from five different chicory cultivars (cv.) were collected for extraction of SL. Fresh leaves and roots from four cultivars of industrial chicory (*Cichorium intybus* L.), cv. “Benulite”, cv. “Goldine”, cv. “Larigot” and cv. “Maestoso” (grown by Sensus, B.V., Roosendaal, The Netherlands); were sampled September 2016 from the test field of Sensus (Colijnsplaat, The Netherlands, 51_575787N, 3_825727E). In addition, a salad-variety of chicory was used for SL optimized extraction: 1) fresh leaves and roots were collected from chicory cv. “Spadona” (DSV Ltd., Denmark) sown as a pure sward (7 kg seeds/ha) in May 2017 and harvested in September 2017 at the experimental farm of the University of Copenhagen (Taastrup, Denmark, 55_6704800N, 12_2907500E). Several whole plants from all five cultivars were harvested. Leaves were picked and frozen to −80 °C, freeze-dried for 48 hours and then dry blended using a kitchen hand blender. Roots were dug up, quickly hand-grinded and frozen at −80 °C, followed by freeze drying. All freeze-dried materials were stored at −20 °C until SL extraction.

### 2.2 Sesquiterpene lactones enriched extraction method for multi-cultivar network

An extraction procedure optimized for recovery of SL was performed as described (Ferioli and D'Antuono, 2012) with modifications. Dry plant material was dissolved in 15 mL of 2% (v/v) formic acid in MeOH/H_2_0 (4/1; v/v), vortexed for 1 minute, sonicated for 10 min at room temperature (RT) and centrifuged at 2647 g for 10 minutes (repeated three times). Extracts were dried under reduced pressure at 35 °C and freeze dried for 30 minutes. Dried extract was recovered with Viscozyme® L cellulolytic enzyme mixture (Sigma-Aldrich) in Citrate-Phosphate buffer pH 5.4, (concentration 10 mg/1 mL buffer). For complete removal of bound sugars, the mixture was left overnight at 37 °C. For collection of metabolites and removal of free sugar, ethyl acetate was added to the aquatic phase and the phases were separated by centrifugation. The collected ethyl acetate phase was dried under reduced pressure (35 °C) and extract was dissolved in 14% methanol in dichloromethane and further purified by solid-phase extraction (SPE). A SPE vacuum manifold was equipped with 12 × 6 mL SPE tubes (Supelclean® LC-Si SPE tubes, Supelco 505374). SPE cartridges were conditioned with 6 mL dichloromethane/i-propanol (1/1;v/v) and equilibrated with 6 mL dichloromethane. The extract was loaded in each SPE tube and the obtained liquid fractions were transferred into one glass flask. Collected fractions were dried under nitrogen flux and the resulting purified extracts were weighed.

Extracts used for *in vitro* assays were suspended in 100% dimethyl sulfoxide (DMSO) at a concentration of 100 mg dry weight extract/mL DMSO and stored at −20 °C until use. Extract used for chemical analysis were suspended in MeOH at a concentration of 10 mg dry weight extract/mL methanol.

### 2.3 Quantification and identification of sesquiterpene lactones for multi-cultivar network

#### 2.3.1 UHPLC-MS QTOF

Ultra-high performance liquid chromatography-high resolution mass spectrometry (UHPLC-HRMS) was used for analysis of all compounds present in the examined extracts. The analysis was performed using an Agilent Infinity 1290 UHPLC system QTOF (Agilent Technologies, Santa Clara, CA, USA) equipped with a diode array detector. The column was an Agilent Poroshell 120 phenyl-hexyl (2.1 × 250 mm, 2.7 μm). The gradient was linear with water (A) and acetonitrile (B) both buffered with 20 mM formic acid, starting at 10% B and increased to 100% in 15 min, and held for 2 min. The flow was 0.35 mL/min and column temperature was 60 °C. Sample injection volume was 1.00 μl. The MS detection was performed in positive atmospheric pressure ionization-electrospray source mode (Agilent 6545 QTOF MS with an Agilent Dual Jet Stream electrospray ion source). The drying gas temperature was 250 °C, the gas flow was 8 l/min, with a sheath gas temperature of 300 °C and flow of 12 l/min. Capillary voltage was set to 4000 V and nozzle voltage to 500 V. Mass spectra were recorded at 10, 20 and 40 eV as centroid data for *m/z* 85–1700 in MS mode and *m/z* 30–1700 in MS/MS mode, with an acquisition rate of 10 spectra/s.

#### 2.3.2 HPLC-MS QQQ

Quantification of SL was performed using an Agilent Infinity 1290 UHPLC system QQQ (Agilent Technologies, Santa Clara, CA, USA) with a diode array detector. The column used was a Phenomenex Kinetex C18 (150*2.1 mm, 2.6 μm). The gradient was linear with water (A) and acetonitrile (B) (both buffered with 20 mM formic acid) starting at 10% B at a flow rate of 0.59 mL/min and ending at 10 min (100% B). The ion source was Agilent Jet Stream Electrospray Ionization with Dynamic MRM as scan type. An injection volume of 1 μL was used. MS detection was performed in positive ion mode on an Agilent 6490 Triple Quad MS equipped with Agilent Dual Jet Stream electrospray ion source with Dynamic MRM as scan type. Identification of individual compounds was based on previous findings (Graziani et al., 2015). Standards used for quantification of SL were lactucin (3809, Extrasynthese SAS, 69727 GENAY Cedex) and 11beta,13-dihydrolactucopicrin (3811, Extrasynthese SAS, 69727 GENAY Cedex). A dilution curve of 0.1, 1, 10 and 100 parts per million (PPM) was used to calculate the equation.

### 2.4 Molecular networks for multi-cultivar network

The molecular network pipeline has previously been reported (Nothias et al., 2018). Briefly, the following procedures were followed:

#### 2.4.1 Data preprocessing with MZmine

LC-MS/MS chromatograms were converted to mzXML files using ProteoWizard's MSConvert (version 3.0)(Kessner et al., 2008) to be preprocessed with MZmine (version 3.7.2) (Pluskal et al., 2010) with the following parameters. Mass detection: MS level 1; Retention time Auto range, Noise level 3000. MS level 2; Retention time Auto range, Noise level 0. ADAP Chromatogram builder (Myers et al., 2017): Retention time Auto range, MS level 1, Min group size 3, Group intensity 10000, Min highest intensity 30000, *m/z* tolerance 0.015 *m/z* or 20 ppm. Chromatogram deconvolution: Wavelets (ADAP), S/N threshold 10, min feature height 10, coefficient/area height 110, peak duration range 0–10, RT wavelet range 0–0.10, *m/z* center calculation MEDIAN, *m/z* range for MS2 scan pairing (Da) 0.015, RT range for MS2 scan pairing (min) 0.3. Isotopic peak grouper: *m/z* tolerance 0.015 *m/z* or 20 ppm, retention time tolerance 1 absolute (min), maximum charge 2, representative isotope most intense. Join aligner: *m/z* tolerance 0.015 *m/z* or 20 ppm, weight for *m/z* 75, retention time tolerance 1 absolute (min), weight for RT 25. Feature list rows filter: minimum peaks in a row 3, minimum peaks in an isotope pattern 2, keep only peaks with MS2 scans (GNPS). Gap-filling, Peak finder: Intensity tolerance 10%, *m/z* tolerance 0.015 m/z or 20 ppm, retention time tolerance 1 absolute (min). A feature table containing all extracted mass spectral features was exported in the .csv format and associated aggregated MS2 fragmentation spectra were exported in the .mgf format and submitted to feature-based mass spectral molecular networking through GNPS.

#### 2.4.2 GNPS molecular network workflow parameters

A molecular network was created using the feature-based mass spectral molecular networking online workflow through the Global Natural Products Social Molecular Networking Platform (GNPS) (Wang et al., 2016; Nothias et al., 2020). The data was filtered by removing all MS/MS fragment ions within ± 17 Da of the precursor m/z. MS/MS spectra were window filtered by choosing only the top 6 fragment ions in the ± 50Da window throughout the spectrum. The precursor ion mass tolerance was set to 0.02 Da and a MS/MS fragment ion tolerance of 0.02 Da. A network was then created where edges were filtered to have a cosine score above 0.70 and more than 5 matched peaks. Further, edges between two nodes were kept in the network if and only if each of the nodes appeared in each other's respective top 10 most similar nodes. Finally, the maximum size of a molecular family was set to 100, and the lowest scoring edges were removed from molecular families until the molecular family size was below this threshold. The spectra in the network were then searched against GNPS′ spectral libraries. The library spectra were filtered in the same manner as the input data. All matches kept between network spectra and library spectra were required to have a score above 0.7 and at least 6 matched peaks. The mass spectral molecular networking job is accessible at: https://gnps.ucsd.edu/ProteoSAFe/status.jsp?task=6fb25560f4b948fa9ac0406fdabdb321.

#### 2.4.3 Statistics and calculation of bioactivity score

The R-based Jupyter notebook retrieved from https://github.com/DorresteinLaboratory/Bioactive_Molecular_Networks) called Bioactive Molecular Networks was used to calculate bioactivity scores for every compound detected in all samples (Nothias et al., 2018). Bioactivity scores are defined as the Pearson correlation scores (r) between feature intensities across samples and the bioactivity levels associated with each sample (Nothias et al., 2018). For each bioactivity score a corresponding p value (r_pval) was calculated and corrected for multiple hypothesis testing using the Bonferroni method. The modified Jupyter notebook is accessible in the Supplementary file S1.

A summary of most predominant putative chemical classes per mass spectral molecular family based on GNPS spectral library hits was created using the MolNetEnhancer workflow (Ernst et al., 2019). Chemical class annotations were performed using the ClassyFire chemical ontology (Djoumbou Feunang et al., 2016). The chemical classes were visualized by manually changing the node colors in CytoScape (version 3.7.2) (Shannon et al., 2003). The GNPS-MolNetEnhancer job is publicly accessible at: https://gnps.ucsd.edu/ProteoSAFe/status.jsp?task=03c8a30784854e84b5902e68bf2871fc.

#### 2.4.4 Prediction of bioactivity score significance and mapping onto the molecular network

The R-based Jupyter notebook used to calculate per feature bioactivity scores outputs a node attribute table. For visualization of bioactive molecular networks in Cytoscape, the Select function in Cytoscape was used to filter nodes based on their Pearson correlation score (r) and the multiple hypothesis corrected p value (r_pval). Only molecules having a bioactivity score above r > 0.80 and a p value of <0.03 were considered significantly associated with bioactivity and represented as a square node in the bioactive molecular networks.

### 2.5 Fractionation and purified compounds

135 g of freeze-dried chicory leaves (*cv.* Spadona) were extracted at room temperature with 500 mL of a mixture DCM:MeOH (1:1) and sonicated for 10 minutes. After filtration, the extraction was repeated twice. Solvents were removed under reduced pressure to lead to a greenish dry extract (6.5 g). The extract was subsequently fractionated into 18 fractions using flash chromatography. The extract was then solubilized in MeOH/DCM and Silica-diol phase (for further dry loading) were added before solvent evaporation using a rotary evaporator. The extract was then submitted to flash chromatography using a 100 g snap cartridge containing diol-functionalized silica (Flow: 50 mL/min) with the following gradient: **1)** Hexane/Iso-propanol: 5% to 100% Iso-propnaol over 25 min, **2)** 100% Isopropanol held for 2 minutes, **3)** Iso-prop/water: 0% to 80% water over 15 min. The cartridge had been previously equilibrated with hexane/Iso-propnaol (95:5) for 5 min. 18 fractions (100 mL each) were collected and the solvents evaporated using rotary evaporator to lead to dry fractions. Fractions 1–11 were tested *in vitro* for anti-parasitic activity using *A. suum* third stage larvae (L3). Fractions 12–18 were too polar to be detected by LC-MS and were excluded from further analysis.

### 2.6 Purification of sesquiterpene lactones

Fractions 5 to 8 were grouped for further fractionation on flash chromatography using a Biotage Cartridge Snap KP-C18-HS 30 g (Ref: FSL0-1118-0030), a H_2_O/MeOH solvent system and a 25 mL/min flow. Dry deposit was realized with mixing 1 g C18 silica to the extract. The following gradient was employed: **1)** 10% to 40% MeOH over 25 min, **2)** 40% to 50% MeOH over 10 min, **3)** 50–100% MeOH over 2 min, **4)** 3 min at 100%. 45 collection tubes were collected but grouped into 17 fractions (F2-A to F2-Q). Fraction F2–H was subjected to reverse-phase HPLC purification (Phenomenex, Luna C18(2) 100 Å 250 × 10 mm, 5 μm) using an isocratic elution with 85% H_2_O–CH_3_CN (+0,1% Formic acid) at a flow rate of 5 mL/min to give lactucin (6 mg, RT = 13 min). Fraction F2–K gave 8 8-deoxy-lactucin (12 mg, RT = 17.5 min) and dihydro-8-deoxy-lactucin (1 mg, RT = 19.5 min) with 77% H_2_O-acteonitrile while fraction F2–N gave lactucopicrin (18 mg, RT = 12 min) with 67% H_2_O–CH_3_CN. In addition, lactucin (Product Code #3809) and 11beta,13-Dihydrolactucin (Product Code #3810) were purchased from EXTRASYNTHESE SAS, France.

### 2.7 LC-MS analysis for fractions and pure compounds

Extract and fractions were solubilized at a concentration of 1 mg/mL in MeOH. Pure compounds were prepared at a concentration of 0.2 mg/mL in MeOH. Ultra-high Performance Liquid Chromatography-High Resolution Mass Spectrometry (UHPLC-HRMS) was performed on a Dionex Ultumate 3000 RS equipped with a diode array detector and coupled to Bruker QTOF Maxis with an electrospray ionization source. The analyses (injection volume: 2 μl) were performed on a reversed-phase column (Phenomenex Luna Omega C-18, 150 × 2.1 mm, 1.6 μm) employing a gradient of 10% to 100% CH3CN in water over 10 min followed by 3 min at 100% CH3CN (all solvents buffered with 0.1% formic acid) with a flow rate of 0.5 mL/min. Analyses were performed in positive ion mode.

### 2.8 NMR of pure compounds

1D-NMR were acquired on a Bruker Avance 800 spectrometer equipped with a 5 mm TCI Cryoprobe. All compounds were dissolved in DMSO-*d*6 (500 μL) at 303 K. All chemical shifts were calibrated on the residual solvent peak (DMSO-*d*6, 2.50 ppm (^1^H) and 39.5 ppm (^13^C)). The spectral data was compared with previously published data (Pyrek, 1985; Seto et al., 1988; Van Beek et al., 1990; Kisiel and Zielińska, 2001).

### 2.9 Molecular networking for fractionation network

#### 2.9.1 Data preprocessing with MZmine for fractionation network

LC-MS/MS chromatograms were converted to mzXML files using ProteoWizard's MSConvert (version 3.0) (Kessner et al., 2008) to be preprocessed with MZmine (3.7.2) with the following parameters. Mass detection: MS level 1; Retention time 1–12 min; Noise level 1000. MS level 2; Retention time 1–12 min; Noise level 300. Chromatogram builder: Retention time 1–12 min; Ms level 1; Min time span 0.01 min; Min height 3000; m/z tolerance 0.01 m/z or 30.0 ppm. Chromatogram deconvolution: Baseline cut-off Min peak height 2000, Peak duration 0.01–3.00, Baseline level 800; *m/z* range for MS2 scan pairing 0.02; RT range for MS2 scan pairing 0.3 min. Isotopic peak grouper: *m/z* tolerance 0.0 *m/z* or 20.0 ppm; Retention time tolerance 0.2 min. Join aligner: *m/z* tolerance 0.0 *m/z* or 30.0 ppm.; Weight for *m/z* 75; Retention time tolerance 0.5 min; Weight for RT 25. Gap filling: intensity tolerance 10.0%; *m/z* tolerance 0.0 *m/z* or 30.0 ppm; Retention time tolerance 0.5 min; RT correction. Feature list rows filter: Minimum peaks in a row 2; Minimum peaks in an isotope pattern 2. Keep only peaks with MS2 scan (GNPS) on. A feature table containing all extracted mass spectral features was exported in the .csv format and associated aggregated MS2 fragmentation spectra were exported in the .mgf format and submitted to feature-based mass spectral molecular networking through GNPS.

#### 2.9.2 GNPS molecular network workflow parameters for fractionation network

A molecular network was created using the feature-based mass spectral molecular networking online workflow through the Global Natural Products Social Molecular Networking Platform (GNPS) (Wang et al., 2016; Nothias et al., 2020). The data was filtered by removing all MS/MS peaks within ± 17 Da of the precursor *m/z*. MS/MS spectra were window filtered by choosing only the top 6 peaks in the ± 50 Da window throughout the spectrum. A network was then created where edges were filtered to have a cosine score above 0.7 and more than 5 matched peaks. Further edges between two nodes were kept in the network if and only if each of the nodes appeared in each other's respective top 10 most similar nodes. The spectra in the network were then searched against GNPS′ spectral libraries. The library spectra were filtered in the same manner as the input data. All matches kept between network spectra and library spectra were required to have a score above 0.7 and at least 6 matched peaks. Precursor ion mass tolerance and fragment ion mass tolerance were set on 0.04 Da.

Bioactivity calculations were performed as described above (Prediction of bioactivity score significance and mapping onto the molecular network). The GNPS-MolNetEnhancer job is publicly accessible at: https://gnps.ucsd.edu/ProteoSAFe/status.jsp?task=b5d1acdf954b43bfba94726e2bec4ed6.

### 2.10 Anti-parasitic studies of chicory extracts, fractions and purified compounds

#### 2.10.1 Ascaris suum *in vitro* mortality assay

Fresh, adult *A. suum* worms were collected at a local slaughterhouse (Danish Crown, Ringsted, Denmark). Embryonation and hatching of eggs was performed as previously described (Williams et al., 2016). Briefly, the eggs were washed and suspended in Hanks’ Balanced Salt Solution (HBSS), and hatched by stirring together with 2 mm glass beads for 30 min at 37 °C. To separate larvae from unhatched eggs, the solution was placed on a 20 μm mesh in a Baermann glass and covered with sterile HBSS and left at 37 °C for 24 hours. The L3 were washed and suspended in larval culture medium (RPMI 1640 supplemented with 2 mM L-glutamine, 100 U/mL penicillin and 100 μg/mL streptomycin) and counted before being used in the assay. To assess mortality, worms were observed after incubation with either test substances or negative control (1% DMSO). Mortality of the worms was determined by the ratio of dead and alive worms which were counted and normalized to negative controls. Dead larvae appeared straight for several seconds whilst live larvae would be moving or in the typical coiled position as previous described (Williams et al., 2016). All analyses were performed in triplicates and counted after 24 hour incubation at 37 °C. SL-enriched semi-purified extracts were investigated at two-fold dilutions between 1000 μg/mL and 62.5 μg/mL, whilst fractions were all tested at 50 μg/mL and pure compounds at two-fold dilutions between 500 μg/mL, and 7.8 μg/mL. Synergistic analysis was performed based on 8-DOL at two-fold dilutions between 500 μg/mL and 15.63 μg/mL combined with either 500 μg/mL LACP or 300 μg/mL LAC (based on the approximate EC_20_ value). Expected additive effects were calculated as follows:

$$Predicted\;additive\;effect==(1-\frac{1-EC50\;of\;8‐DOL}{100})*(1-\frac{1-(\;Mortalilty\%\;of500\frac{\mu g}{mL}LAC\;or300\frac{\mu g}{mL}LAC}{100})*100$$

#### 2.10.2 Teladorsagia circumcincta larval mortality assay (LMA)

*Teledorsagia circumcincta* eggs were collected from feces from two Merino lambs experimentally infected one month before with a susceptible field strain isolated in a previous study and maintained in the laboratory (Martínez-Valladares et al., 2012). This infection was approved by the Animal Ethics Committees of the Instituto de Ganadería de Montaña, Spanish Council of Research (CSIC) and Junta de Castilla y León, following the national regulations (R.D. 53/2013). Eggs were purified from freshly collected feces by sieving and centrifugation. Eggs were added to a large glass with two filters and left to incubate for 24 hours at 23 °C. The next day, the hatched L1 were recovered. All extracts were screened at concentration of 1 mg/mL and 1% DMSO in triplicates in 96 well plates in 2 adjacent days with different larval cultures. DMSO (1%) was used as a negative control. The assay was read after 48 hours incubation. Moving or coiled-up larvae were counted as alive, and immobile and completely straight larvae as dead. The results were corrected with the negative controls using the formula:

$$\text{\%Mortality}=\;\left(1-\frac{\text{\%survival of L}1\text{ larvae exposed to chicory extract}}{\text{\% survival of negative controls}}\right)$$

EC_50_ calculations were made using 2-fold dilutions, ranging from 1000 μg/mL to 62.5 μg/mL. The same controls were used as in the mortality assay.

#### 2.10.3 Cooperia oncophora larval development assay (LDA)

The *C. oncophora* drug-susceptible isolate used has previously been described (Al Gusbi et al., 2014). Eggs were purified from two Holstein-Friesian calves infected with 40,000 L3 at an age of four months. Infection of animals was approved by the Landesamt für Gesundheit und Soziales (LAGeSo) under the reference number H0337/17. Between 4 and 6 weeks post infection, feces were collected rectally and eggs were purified on a sugar step gradient (Demeler et al., 2010). Eggs were concentrated to approximately 5–6 eggs/μl. Assays were conducted in 48 well plates in a total volume of 300 μl containing 200 μl distilled water, 30 μl of drug solutions and 50 μl growth medium. The latter consisted of a 2:2:1 mixture of Yeast/Earle's extract (1% yeast (w/v) in 0.9% NaCl, diluted 1:10 in Earle's solution (Sigma-Aldrich), 0.3 mg/mL Amphotericin B (Sigma-Aldrich) and 1.5 mg/mL lyophilized *Escherichia coli* K12 (in distilled water, autoclaved). Finally, 20 μl egg suspension were added to each well. The final concentration of extracts were in the range of 500–7.66 μg/mL (two-fold serial dilutions). DMSO concentration in all wells was 0.5%. Plates were sealed with Parafilm and incubated at 25 °C for 7 days before L3 and earlier stages (eggs + L1 + L2) were counted separately. Plates with less than 80% development in the negative control wells were discarded. For each extract and concentration, at least 6 replicates from 3 different plates were included in the final analysis.

### 2.11 Statistical analyses

EC_50_ values were calculated by by non-linear (least squares) regression using the model log (inhibitor) vs. response - variable slope (four parameter) in GraphPad Prism (v7). To assess the effect of different cultivars and tissue type (root or leaf) on *A. suum* mortality EC_50_ values, a two-way ANOVA was performed with significance taken at *P* < 0.05, and post-hoc testing carried out using Fisher's L.S.D. Differences between EC_50_ values in the *C. oncophora* LDA were computed using the extra sum-of-squares F- test.

## 3 Results and discussion

Chicory is a widely-bred plant with many cultivars that are utilized for different purposes. Chicory that is used as forage and for salad consumption as has been bred for high nutritive value in the leaves. In contrast, cultivars used for inulin production have been bred for high inulin content in the roots with less attention placed on the chemical composition of the leaves. Thus, considerable variation potentially exists in the chemical profile of different cultivars. To encompass this variation, we obtained extracts from the salad cultivar ‘Spadona’, and four different inulin-producing cultivars. These extracts were then investigated for their anti-parasitic effects and analysed by UHPLC for metabolites such as SL.

### 3.1 Molecular network analysis of the anti-parasitic activity of chicory cultivars against *A. suum*

Earlier studies have suggested that the SL may be responsible for the anthelmintic activity (Williams et al., 2016; Peña-Espinoza et al., 2020). In order to pursue this question, we first used samples from cv. Spadona, which we have previously shown to be active against *A. suum*, and confirmed that SL-enriched semi-purified extracts had more potent activity than crude extracts of the same leaf material. Crude (100% MeOH) Spadona extract was compared to that of a semi-purified extract that had been subjected to solid-phase extraction (SPE) using a C14 column, resulting in a twofold increase of total SL. When tested for anti-parasitic activity against *A.* suum (see below), the crude extract had an EC_50_ of 2648 μg/mL, whilst the semi-purified extract had an EC_50_ of 170 μg/mL, indicating an almost 16-fold increase in activity due to the extraction process. Thus, semi-purified extracts by SPE were used for the subsequent screening and network analysis.

**Fig. 1:**
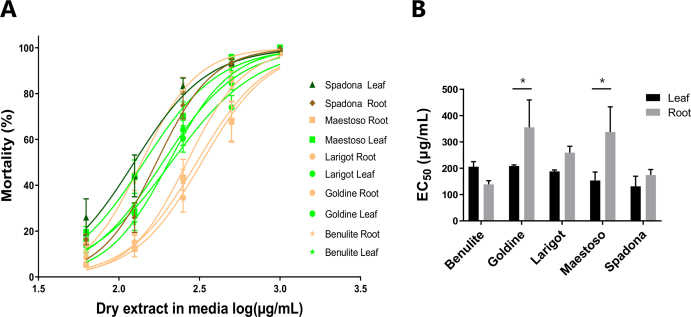
**Dose-dependent anti-parasitic effects of chicory extracts A)** Concentration-response curve of chicory extracts on mortality of Ascaris suum third stage larvae. The inhibition is calculated relative to the control (1% DMSO, corresponding to the amount of DMSO in the samples). The means are presented with S.E.M., based on three different extractions, each tested in triplicate in the A. suum assay. Colors are used to demonstrate the trend of differences between the leaf extracts (green lines and markers) and the root extracts (orange lines and markers). **B)** EC_50_ values of leaf and root extracts from different cultivars. Three independent extractions were made and the EC_50_ calculated for each (mean of N = 3 with S.E.M. is presented). The effects of tissue type and cultivar were assessed by two-way ANOVA. *P < 0.05 by Fisher's L.S.D. (For interpretation of the references to color in this figure legend, the reader is referred to the Web version of this article.)

We obtained semi-purified extracts (in triplicate) from leaf and root material from four inulin cultivars and from cv. Spadona. This resulted in 30 samples, that all were tested for anti-parasitic activity against the *A. suum* using a mortality assay that we have previously shown to be a robust and reproducible means of assessing anti-parasitic activity of plant extracts (Williams et al., 2016). The concentration-response curves of each extract indicated a clear, concentration-dependent mortality (Fig. 1A). There was a significant effect of plant tissue type on EC_50_ values (*P* < 0.05 by two-way ANOVA). In cv. Goldine and cv. Maestoso, the EC_50_ was lower in the leaf extract than the root extract (*P* < 0.05 by Fisher's L.S.D.), whereas there were not significant differences between the tissue extracts for the other cultivars (Fig. 1B). Cultivar did not significantly influence EC_50_ (*P* = 0.08 by two-way ANOVA), however cv. Spadona displayed the most potent anti-parasitic effect against *A. suum*.

**Fig. 2:**
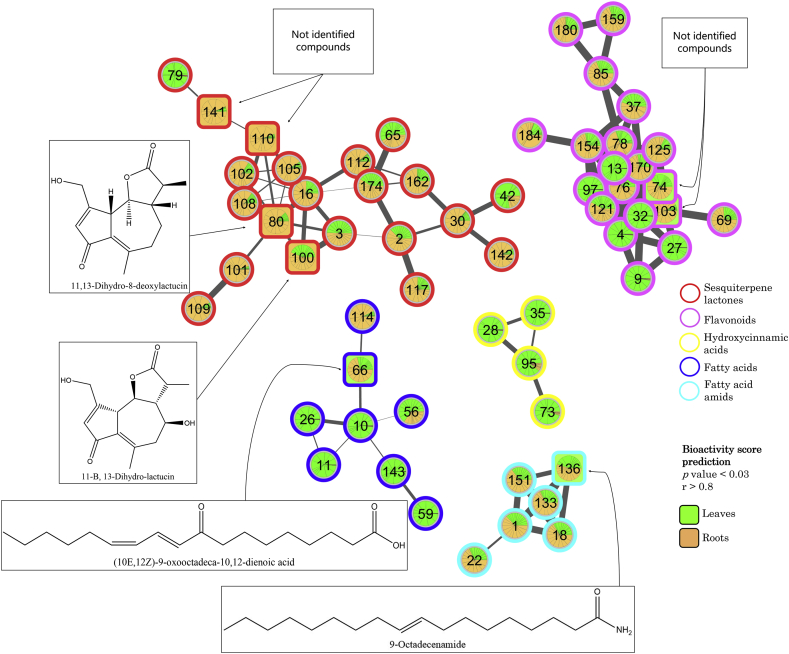
**Chicory cultivar molecular network**. The GNPS MolNetEnhancer workflow highlighted five different molecular families, including sesquiterpene lactones, flavonoids, hydroxycinnamic acids, fatty acids and fatty acid amids of which four contained features significantly associated with the inhibitory effect against A. suum. Each node is marked with a number corresponding to the feature ID and respective parent mass (see Supplementary File 2), a pie chart indicating the relative amount of the compound distributed in inulin-cultivar leaves (green, n = 12) and roots (brown, n = 12). Square nodes indicate compounds with a high bioactivity score (P < 0.03, r > 0.8); only the four inulin cultivars were used for this calculation. Features annotated through GNPS spectral library matching with a high bioactivity score are indicated with structure images and arrows. High bioactivity score compounds with no match in the GNPS spectral library are marked with a question mark. All known compounds were annotated using the GNPS spectral library databases as described in Supplementary Table 1. (For interpretation of the references to color in this figure legend, the reader is referred to the Web version of this article.)

In order to predict the likely active compound groups in the extracts, we performed feature-based molecular networking using GNPS (Wang et al., 2016; Nothias et al., 2020) in combination with the MolNetEnhancer workflow (Ernst et al., 2019). A combined network, illustrating features most strongly associated with activity against *A. suum* (square nodes) and with the most predominant chemical classes (per molecular family) highlighted is shown in Fig. 2. The network illustrates the structural relationship between each feature based on their MS/MS spectral similarity, where high spectral similarity represents high chemical structural similarity. Connected nodes in the network show a MS/MS spectral similarity of >0.7 and thus represent chemically structurally highly similar features. The MolNetEnhancer workflow summarizes most predominant compound classes per molecular family (connected component of a graph) found through GNPS spectral library matching. Most predominant compound classes according to the ClassyFire ontology at the direct parent level (CF_Dparent) are highlighted with designated colors (circles). Finally, each feature was designated with a calculated bioactivity score (Nothias et al., 2018). For bioactivity calculations, only the four inulin cultivars were included as they had closely related chemical profiles, hence making them good candidates for detecting compounds related to their bioactivity.

The most predominant chemical classes could be retrieved for five molecular families based on the fragmentation spectra and comparison to the GNPS spectral libraries including SL, hydroxycinnamic acids, fatty acids, fatty acid amides and flavonoids. Examination of the GNPS spectral library matches, together with the calculated bioactivity scores, allowed us to evaluate which features could be responsible for the observed activity. Nine features had a bioactivity score with a *P* value < 0.03 and r > 0.8: four of these were SL, two were flavonoids, one was a fatty acid, one a fatty acid amid and the last compound could not be assigned to any of the five groups (Table S1). The finding that the majority of the predicted bioactive compounds were SL supports previous findings suggesting that these may be the primary anti-parasitic compounds in chicory (Peña-Espinoza et al., 2020).

### 3.2 Bio-guided fractionation and bioactivity-based molecular networks

**Fig. 3:**
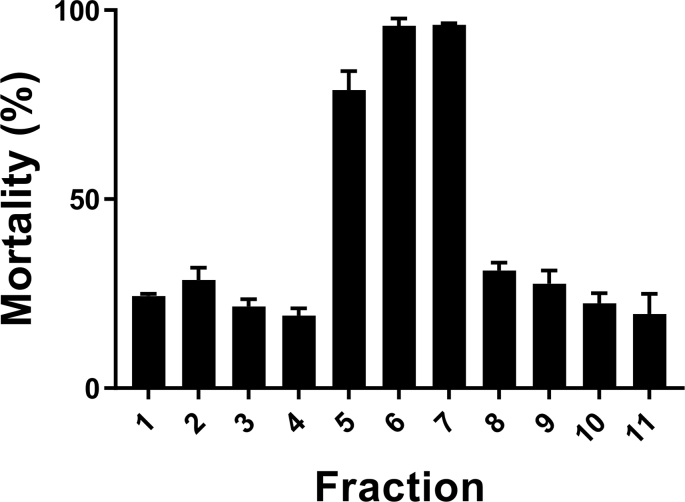
**Anti-parasitic effects of chicory fractions**. Percentage mortality effects of fractions from chicory (cv. Spadona) elucidated from flash chromatography on *Ascaris suum* third stage larvae. Mortality was calculated relative to the negative controls. N = 3 and means are presented with error bars (S.E.M.).

Whilst SL were the predominant compounds with high bioactivity scores, other groups of compounds also showed a significant predicted activity (Fig. 2). This may indicate that other compounds in chicory also have strong anti-parasitic activity, or rather that the concentrations of these compounds correlate with those of the SL, and thus tend to cluster together in the network. To distinguish between these possibilities, we proceeded with bio-guided fractionation to conclusively determine the active compounds. We selected the Spadona leaf sample with the lowest EC_50_ for further bio-guided fractionation. Each fraction was tested for activity against *A. suum*. Fraction 5, 6 and 7 induced mortalities of 70–96% whereas other fractions induced only low mortalities (≤30%; Fig. 3).

**Fig. 4:**
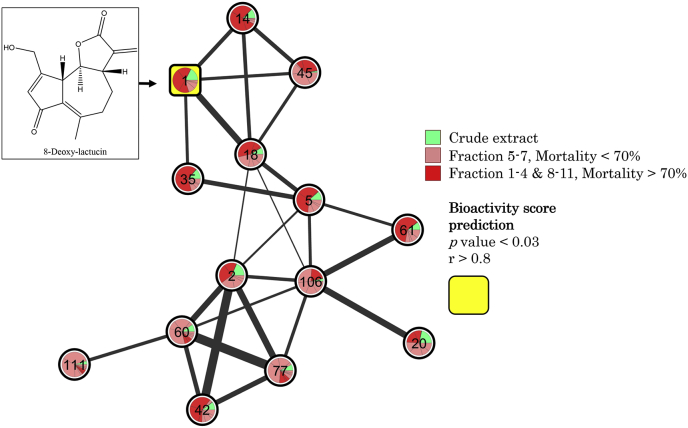
**Chicory fraction molecular network**. Bioactivity-Based Molecular Network of sesquiterpene lactones in all fractions from chicory (cv. Spadona), as listed in Fig. 3. Each node is marked with a number corresponding to the feature ID and the respective precursor mass (see Supplementary File 3), a pie chart indicates the amount of the compound distributed in fractions designated by colors corresponding to the level of mortality (%) presented in Fig. 3. The square node indicates a high bioactivity score (P < 0.03, r > 0.8). (For interpretation of the references to color in this figure legend, the reader is referred to the Web version of this article.)

Fractions 5–7 were analysed by LC-MS, which showed that SL were the major constituents in all three fractions, thus indicating that they are primarily responsible for the anti-parasitic activity of chicory. Next, GNPS was used to create a molecular network based on all fractions, as described above. This showed 8-DOL to be the only compound with a high predicted bioactivity score (Fig. 4), indicating that this compound is the major anti-parasitic compound. As 8-DOL and dihydro-8-DOL are very similar in size and charge, the two peaks were overlapping in the chromatogram raw data. This could potentially result in one or the other being falsely up-regulated by the other peak. For this reason, and to unequivocally identify the active SL in chicory, each individual SL was purified.

**Fig. 5:**
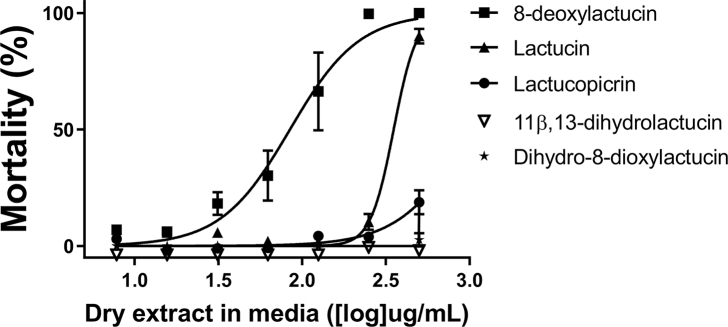
**Anti-parasitic effects of purified sesquiterpene lactones.** Concentration-response curve of purified sesquiterpene lactones on mortality of Ascaris suum third stage larvae. Concentrations ranged from 250 μg/mL. N = 3 and means are presented with error bars (S.E.M.).

Fractions 5–7 (containing LAC, 8-DOL, dihydro-8-DOL, and LACP) were pooled and used to purify compounds for further analysis of activity against *A. suum*. LAC, 8-DOL, dihydro-8-DOL and LACP were successfully purified and their identity determined by NMR that matched previously published spectral data. We also noted in our cultivar network (Fig. 2) that dihydro-8-DOL and 11,13-dihydro-LAC were also predicted to have high activity. As 11,13-dihydro-LAC was not present in fractions 5–7, we purchased this commercially. Concentration-response-curves and EC_50_ values clearly showed 8-DOL to be the primary active compound, with an EC_50_ of 85 μg/mL, whilst LAC and LACP only exhibited low activity (Fig. 5).

This corresponds with the calculated bioactivity score based on the fractions (Fig. 4) that suggested 8-DOL to be the only active compound. Dihydro-8-DOL and 11,13-dihydro-LAC did not exhibit any activity against *A. suum* at the highest concentration (500 μg/mL). LAC and LACP did show concentration-dependent activities against *A. suum*, however these were substantially lower than 8-DOL, with EC_50_ values of 352 μg/mL and 1028 μg/mL, respectively. This indicates that LAC and LACP do not possess significant anti-parasitic properties in isolation. However, as all the compounds are present together in the plant tissue, the possibility exists that different compounds may interact to exert a synergistic effect.

### 3.3 Synergistic/additive anti-parasitic effects of sesquiterpene lactones

**Fig. 6:**
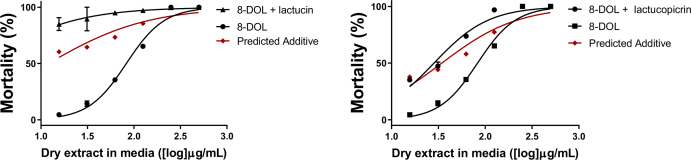
**Synergistic effects of sesquiterpene lactones against Ascaris suum.** Synergy analysis of 8-deoxylactucin (8-DOL) with 300 μg/mL lactucin **(A)** and B) 500 μg/mL lactucopicrin **(B)** evaluated in the *Ascaris suum* mortality assay. 8-DOL concentrations started at 15.6 μg/mL and incrementally doubled until reaching 500 μg/mL 8-DOL was analysed alone at increasing concentrations and together with a fixed concentration of other compounds. In red, the predicted additive effect is illustrated. This was calculated based on the measured activity of 300 μg/mL lactucin and 500 μg/mL lactucopicrin alone. N = 3 and means are presented with error bars (S.E.M.). (For interpretation of the references to color in this figure legend, the reader is referred to the Web version of this article.)

Although our studies revealed 8-DOL to be the primary anti-parasitic entity, any effect of a bioactive plant may stem from a range of interacting compounds. The activity of 8-DOL at a range of concentrations was thus analysed in combination with LAC and LACP (EC_20_ concentrations) to evaluate possible additive or synergistic effects. The combined effect of 8-DOL and LAC exceeded the predicted additive effect against *A. suum*, indicating a synergistic interaction between these compounds (Fig. 6A). In contrast, the combined effect of 8-DOL and LACP did not exhibit a higher effect than the predicted additive effect (Fig. 6B). Thus, whilst LAC did not possess significant activity in isolation, it may potentiate the effect of 8-DOL, suggesting a synergistic mechanism for the activity of the whole chicory plant.

### 3.4 Chicory has broad-spectrum activity against a range of parasitic nematodes

**Fig. 7:**
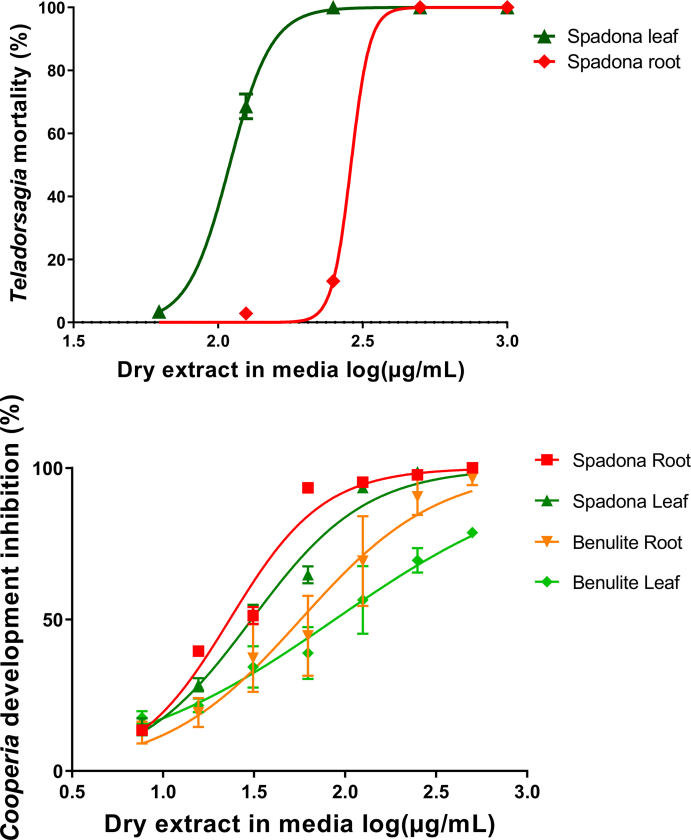
**Anti-parasitic activity of chicory extracts against ruminant nematodes**. Concentration-response curves obtained for A) Teladorsagia circumcincta (mortality of first stage larvae (L1)) after incubation with cv. Spadona leaf and root extract. Each point on the curve is the mean of the bioassay replicates, n = 3; and error bars represent S.E.M. based on means of replicates from the activity assays only. B) Cooperia oncophora larval development assay (LDA). The inhibition of development was calculated relative to the control (0.5% DMSO, corresponding to the amount of DMSO in the samples). Each point on the curve is the mean of the bioassay replicates, n = 6; and error bars represent S.E.M. based on means of replicates from the activity assays only.

In order to assess whether the effects observed against *A. suum* were also relevant to other helminths of socioeconomic importance, we tested a selected number of samples against two ruminant helminths. In livestock production, chicory has been most commonly used as a forage for grazing ruminants, and we therefore assessed the activity against nematodes of sheep, *T. circumcincta* and cattle, *C. oncophora* (Fig. 7). We found that semi-purified Spadona leaf and root extracts induced significant mortality in *T. circumcincta* larvae, similar to that observed with *A. suum* (Fig. 7A)*.* Moreover, a variety of samples inhibited the development of *C. oncophora* larvae (Fig. 7B), confirming our earlier observations with different *in vitro* assays (Peña-Espinoza et al., 2017). Importantly, a similar pattern of extract efficacy suggests that the same combination of compounds was responsible for the effect against *C. oncophora* as in *A. suum*. Leaf and root material from cv. Benulite was also tested in the LDA against *C. oncophora*, and again showed same trend as for *A. suum*, where we observed that the root extract had a lower EC_50_ value than the leaf extract (*P <* 0.05 by extra sum-of-squares F-test; Fig. 7B).

Taken together, these data conclusively show for the first time that the anti-parasitic activity of chicory derives from SL. These compounds are well known for their biological activity, and have been proposed to as a promising natural resource for development of new drugs against a range of infectious diseases. Our results provide an impetus to further develop chicory as a preventive or therapeutic measure against parasitic infection, either by targeted selection of SL-rich cultivars or production of pure single compound(s) that may prove efficacious against a wide range of nematode species in both animals and humans.

A clear finding in this study is that the activity of SL, whilst predominantly attributable to 8-DOL, appeared to work in a synergistic fashion involving at least two different compounds. This suggests that the most appropriate use of chicory as an anthelmintic therapy may be in the form of the whole plant or as targeted extracts, rather than as a resource for purification of SL, as is done for the production of the anti-malarial drug artemisinin (Martino et al., 2019). As chicory is already widely used as a grazing crop for livestock, this preventative approach may be easily integrated into veterinary medicine. The identification of the active compounds will now for the first time make it possible for plant breeders to selectively breed for cultivars with a high content of the active SL and thus create a cultivar designed for parasite treatment. Whilst our current results were obtained against larval stages, we have previously shown that SL-rich chicory extracts also have strong activity against adult worms from *O. ostertagi* and *C. oncophora* (*ex situ*) suggesting that the SL may broadly target different life-cycle stages (Peña-Espinoza et al., 2015, 2017). Importantly, we have demonstrated that the activity of chicory appears to be conserved across multiple important clades of nematodes that infect ruminants and pigs, as well as humans.

For now, the mode of action of the SL we have identified here remains unknown. Identification of the mechanistic basis of the anti-parasitic activity is a high priority, in order to further optimize the use of chicory or other SL-rich plants as parasite-control options. SL contain a functional α-methylene group that will efficiently react with thiol groups found in free cysteine or cysteine-containing amino acids or peptides *via* a Michael-addition reaction (Simonsen et al., 2013). Neutralization of cysteine-containing molecules (e.g. glutathione) has been proposed as a mechanism underlying the activity of SL against protozoan parasites, whereby free glutathione is prevented from binding to reactive oxygen species, resulting in oxidative stress and parasite necrosis (Barrera et al., 2013; Zimmermann et al., 2013). Whether similar mechanisms operate against helminths remains to be explored.

In conclusion, based on both molecular networking and bio-guided fractionation, we have elucidated the compounds responsible for the anthelmintic properties of chicory. Our results confirm previous studies which hypothesized a role for SL, and we have demonstrated 8-DOL as the primary anti-parasitic compound. Finally, the observed synergistic and additive effects of LAC and LACP, suggests that the anthelmintic effect is not caused by 8-DOL alone but from a combination of several compounds. Future research should focus on harnessing this knowledge to design a new generation of anthelmintic treatments for human and veterinary use.

## Declaration of competing interest

M de Roode is an employee of Sensus B/V. The other authors have no financial or other conflicts of interest.
