# Supplementary material for: Identification of compounds responsible for the anthelmintic effects of chicory (Cichorium intybus) by molecular networking and bio-guided fractionation
Source: Int J Parasitol Drugs Drug Resist. 2021 Feb 11;15:105–14. doi: 10.1016/j.ijpddr.2021.02.002 (PMC7907819; doi:10.1016/j.ijpddr.2021.02.002)
Supplement: Multimedia component 1 [file mmc1.pdf]

**Supplementary Information**

**Valente *et al.***

Supplementary Table 1

Supplementary File 1

Supplementary File 2

Supplementary File 3

Supplementary Table 1. Compounds found in all SL enriched extracts annotated using GNPS spectral library matching through the feature-based molecular networking workflow.

| **GNPS predicted Compound** | **SpecMZ** | **GNPS Library m/z** | **Molecular formular** | **m/z error [PPM]** | **Cosine score** | **X= P-val < 0,03 r > 0,8** |
|---|---|---|---|---|---|---|
| Dehydrocostus lactone | 231.1382 | 231.14 | $C_{15}H_{18}O_2$ | 0 | 0.76 | - |
| 8-Deoxy-lactucin | 261.1131 | 261.11 | $C_{15}H_{16}O_4$ | 3 | 0.92 | - |
| 11-B,13-Dihydro-lactucopicrin | 413.1573 | 413.16 | $C_{23}H_{24}O_7$ | 1 | 0.79 | - |
| 11-B, 13-Dihydro-8-deoxy-lactucin | 263.1278 | 263.13 | $C_{17}H_{18}O_7$ | 4 | 0.91 | x |
| Lactucin | 277.1079 | 277.11 | $C_{15}H_{16}O_5$ | 0 | 0.95 | - |
| Lactucopicrin | 411.145 | 411.14 | $C_{23}H_{22}O_7$ | 0 | 0.95 | - |
| 11-B,13-Dihydro-lactucin | 279.1231 | 279.12 | $C_{15}H_{20}O_5$ | 0 | 0.97 | x |
| Apigenin | 271.0606 | 271.06 | $C_{15}H_{10}O_5$ | 1 | 0.83 | - |
| Luteolin | 287.0557 | 287.06 | $C_{15}H_{10}O_6$ | 2 | 0.76 | - |
| Kaempfeol | 287.0557 | 287.06 | $C_{15}H_{10}O_6$ | 1 | 0.79 | - |
| 9-Oxo-10E,12Z-octadecadienoic | 295.2272 | 295.23 | $C_{18}H_{30}O_3$ | 4 | 0.89 | x |
| 9(10)-EpOME | 279.2319 | 279.23 | $C_{18}H_{32}O_3$ | 3 | 0.88 | - |
| Isochlorogenic acid A | 499.1236 | 499.12 | $C_{16}H_{18}O_9$ | 1 | 0.81 | - |
| trans-5-O-Caffeoylquinic acid | 355.1026 | 355.10 | $C_{16}H_{18}O_9$ | 21 | 0.99 | - |
| Caffeic acid | 181.0501 | 181.05 | $C_9H_8O_4$ | 2 | 0.97 | - |
| p-Coumaric acid | 165.0559 | 165.06 | $C_9H_8O_3$ | 5 | 0.98 | - |
| 9-Octadecenamide | 282.2794 | 282.28 | $C_{18}H_{35}NO$ | 1 | 0.81 | x |
| 13-Docosenamide | 338.3434 | 338.34 | $C_{22}H_{43}NO$ | 7 | 0.91 | - |
| Hyperoside | 465.1035 | 465.10 | $C_{21}H_{20}O_{12}$ | 1 | 0.96 | - |
| Betulin | 443.3882 | 443.39 | $C_{30}H_{50}O_2$ | 0 | 0.86 | - |
| Tributyl phosphate | 555.2927 | 555.29 | $C_{12}H_{27}O_4P$ | 2 | 0.94 | - |
| Chlorogenic acid | 377.0847 | 377.08 | $C_{16}H_{18}O_9$ | 1 | 0.86 | - |
| Chicoric acid | 163.0396 | 163.04 | $C_{22}H_{18}O_{12}$ | 3 | 0.92 | - |
| 6,7-Dihydroxycoumarin | 179.0355 | 179.03 | $C_9H_6O_4$ | 8 | 0.88 | - |
| Quercetin 3-O-malonyl glucoside | 551.1033 | 551.10 | $C_{24}H_{22}O_{15}$ | 0 | 0.95 | x |
| Pheophytin A | 871.5749 | 871.59 | $C_{55}H_{74}N_4O_5$ | 20 | 0.72 | - |

*Supplementary File 1. R-script including used Jupyter notebook modifications. Original script retrieved from Nothias et al., 2018 (https://github.com/DorresteinLaboratory/Bioactive_Molecular_Networks).*

```
# Load and inspect the MZmine feature table with bioassay results
# Change the name in the code below if needed (.CSV file in red)
# NB: Make sure to add the value of bioactivity in the second row.
in_tab <- read.csv("191017SENSUS_RootsBigTen_3_2_ms2_quant_bioactivity_newcal.csv",
stringsAsFactor=FALSE, check.names=FALSE)
dim(in_tab)
in_tab[1:5,]

# Transpose and format column and row labels to follow the workflow below
# Change the the 'BioactivityCHIKV' variable in red below to the column row index name
# corresponding to the bioassay results
tab <- t(in_tab[,-c(1:3)])
tab <- data.frame(Sample_name=sub("\\.mzXML Peak area", "", rownames(tab)), tab)
colnames(tab)[-1] <- c('BioactivityCHIKV', apply(in_tab[,2:3][-1,], 1, paste, collapse='_'))
rownames(tab) <- NULL

# Display the table
tab[1:5,1:5]

# Take out blank rows in the table
if(any(is.na(tab[,2]))) tab <- tab[!is.na(tab[,2]),]

# Add 1 to all to help scaling feature intensities and Normalize the features by TIC
#
tab2 <- tab
tab2[,-c(1:2)] <- t(apply(tab2[,-c(1:2)], 1, function(x) (x+1)/sum((x+1))))

# Calculate the correlation coefficient between a single feature and the bioactivity.
# Scale should help correlation - https://www.ncbi.nlm.nih.gov/pmc/articles/PMC1534033/
cor.test(scale(tab2[,2])[,1], scale(tab2[,3])[,1])[c("estimate", "p.value")]

# How to do for all features
ct <- t(sapply(3:ncol(tab2), function(x) unlist(cor.test(scale(tab2[,2])[,1], scale(tab2[,x])[,1])[c("estimate",
"p.value")])))

# Show the dimensions of the features_quantificaton_matrix.csv
dim(tab2)
dim(ct)


# Create an output table with correlation coefficient value and p-value for every features

ct <- rbind(c("cor"," p_value"), c(0,0), ct)

tab3 <- rbind(t(ct),  as.matrix(tab2))
rownames(tab3) <- NULL
```

```
tab3[1:5, 1:5]
write.csv(tab3, "191021_SensusRoots_features.csv", row.names=FALSE)

# Tranpose the table for molecular networking mapping in Cytoscape
new = t(tab3)
colnames(new) = new[1,]
new = new[-1,]
new = cbind(0:(nrow(new)-1), rownames(new), new)
rownames(new) <- NULL
colnames(new)[1:2] <- c("shared name", "IDs")
new[1,1] <- ""
new[1:5,1:5]
write.csv(new, "191021_SensusRoots_features_correlation.csv", row.names=FALSE)

# Get the significant correlation coefficients for both cases (>0.05)
which(as.numeric(ct[-c(1,2),2])<0.05)

# Show the features ID with correlation coefficient
nm <- colnames(tab)
nm[-c(1:2)][as.numeric(ct[-c(1,2),2])<0.05]

# Call the ID
which(p.adjust(as.numeric(ct[-c(1:2),2]), method = "bonferroni")<0.05)

# Features passing Bonferronii method
nm[-c(1:2)][which(p.adjust(as.numeric(ct[-c(1:2),2]), method = "bonferroni")<0.05)]


# Prepare the new table
new <- cbind(new[,1:5], c(0, p.adjust(as.numeric(ct[-c(1:2),2]), method = "bonferroni")), new[,-c(1:5)])
colnames(new)[6] <- "p_value_corrected"
new[,1:10]

# Write the final table with corrected p_value
write.csv(new, "191021_SensusRoots_pvalue_corrected.csv", row.names=FALSE)
```

**Supplementary File 2**

| Name | Parent mass | Compound_Name |
|---|---|---|
| 1 | 338.3434 | Spectral Match to 13-Docosenamide; (Z)- from NIST14 |
| 2 | 411.145 | Lactucopicrin |
| 3 | 277.1079 | Lactucin |
| 4 | 287.0557 | Luteolin |
| 5 | 203.0318 | |
| 6 | 643.5271 | |
| 7 | 179.0355 | ReSpect:PT112500 6;7-Dihydroxycoumarin\|Esculetin\|Cichorigenin\|Aesculetin\|6;7-dihydroxy-2-benzopyrone \|Esculetol\|Cichoriin aglycon\|6;7-Dihydroxy-2H-1-benzopyran-2-one\|Esculin aglycon\|6;7-dihydroxychromen-2-one |
| 8 | 429.3731 | |
| 9 | 287.0557 | Massbank:PB000166 Kaempferol\|3;5;7-trihydroxy-2-(4-hydroxyphenyl)chromen-4-one |
| 10 | 277.2166 | |
| 11 | 273.1849 | |
| 12 | 209.1538 | |
| 13 | 295.1673 | |
| 14 | 721.5076 | |
| 15 | 637.3064 | |
| 16 | 261.1131 | 8-Deoxy-lactucin |
| 17 | 861.5369 | |
| 18 | 310.3114 | |
| 19 | 465.1035 | hyperoside |
| 20 | 207.1383 | |
| 21 | 792.5613 | |
| 22 | 675.6778 | Spectral Match to 13-Docosenamide; (Z)- from NIST14 |
| 23 | 827.529 | |
| 24 | 764.5355 | |
| 25 | 937.5864 | |
| 26 | 249.1849 | |
| 27 | 317.0666 | |
| 28 | 355.1026 | trans-5-O-Caffeoylquinic acid |
| 29 | 659.2876 | |

| | | |
|---|---|---|
| 30 | 483.129 | |
| 31 | 519.1264 | |
| 32 | 271.0606 | Massbank:PR100224 Apigenin\|Apig\|4';5;7-trihydroxyflavone\|Apigenol\|Chamomile\|5;7-Dihydroxy-2-(4-hydroxyphenyl)-4-benzopyrone\|Naringenin Chalcone |
| 33 | 613.4823 | |
| 34 | 429.3727 | |
| 35 | 499.1236 | NCGC00169984-03!(3R;5R)-3;5-bis[[(E)-3-(3;4-dihydroxyphenyl)prop-2-enoyl]oxy]-1;4-dihydroxycyclohexane-1-carboxylic acid |
| 36 | 343.1533 | |
| 37 | 409.1627 | |
| 38 | 708.5116 | |
| 39 | 613.4825 | |
| 40 | 593.2761 | |
| 41 | 654.3324 | |
| 42 | 497.1442 | |
| 43 | 989.5784 | |
| 44 | 357.1696 | |
| 45 | 691.3139 | |
| 46 | 551.1033 | Quercetin 3-O-malonylglucoside |
| 47 | 685.4361 | |
| 48 | 1342.9281 | |
| 49 | 431.1675 | |
| 50 | 163.0396 | Spectral Match to Chicoric acid from NIST14 |
| 51 | 443.3882 | betulin |
| 52 | 813.5127 | |
| 53 | 389.17 | |
| 54 | 799.3162 | |
| 55 | 954.6156 | |
| 56 | 279.2319 | Spectral Match to 9(10)-EpOME from NIST14 |
| 57 | 797.5181 | |
| 58 | 299.1974 | |
| 59 | 423.3607 | |
| 60 | 716.5677 | |
| 61 | 638.5701 | |
| 62 | 387.1809 | |

| | | |
|---|---|---|
| 63 | 663.4545 | |
| 64 | 1347.8836 | |
| 65 | 375.1807 | |
| 66 | 295.2272 | Spectral Match to 9-Oxo-10E;12Z-octadecadienoic acid from NIST14 |
| 67 | 799.5318 | |
| 68 | 377.0847 | Spectral Match to Chlorogenic acid from NIST14 |
| 69 | 349.199 | |
| 70 | 680.4808 | |
| 71 | 611.5009 | |
| 72 | 732.5622 | |
| 73 | 165.0559 | Spectral Match to p-Coumaric acid from NIST14 |
| 74 | 317.2091 | |
| 75 | 932.638 | |
| 76 | 376.3188 | |
| 77 | 833.5892 | |
| 78 | 409.1705 | |
| 79 | 191.143 | |
| 80 | 263.1278 | Dihydro-8-deoxy-lactucin |
| 81 | 738.549 | |
| 82 | 651.4579 | |
| 83 | 404.2076 | |
| 84 | 2590.1739 | |
| 85 | 795.3355 | |
| 86 | 754.5428 | |
| 87 | 568.4572 | |
| 88 | 316.2121 | |
| 89 | 975.5665 | |
| 90 | 327.2167 | |
| 91 | 629.4761 | |
| 92 | 695.359 | |
| 93 | 629.5105 | |
| 94 | 1073.7337 | |
| 95 | 181.0501 | caffeic acid-emf |

| | | |
|---|---|---|
| 96 | 871.5753 | Pheophytin A |
| 97 | 297.1832 | |
| 98 | 939.6016 | |
| 99 | 987.5654 | |
| 100 | 279.1231 | 11-B;13-Dihydro-lactucin |
| 101 | 249.1486 | |
| 102 | 231.1382 | MoNA:3475533 Dehydrocostus lactone |
| 103 | 351.2145 | |
| 104 | 555.2927 | Spectral Match to Tributyl phosphate from NIST14 |
| 105 | 233.1535 | |
| 106 | 679.438 | |
| 107 | 211.1332 | |
| 108 | 249.1482 | |
| 109 | 249.1484 | |
| 110 | 251.1643 | |
| 111 | 559.5174 | |
| 112 | 333.098 | |
| 113 | 1369.7209 | |
| 114 | 229.1437 | |
| 115 | 570.5091 | |
| 116 | 515.3913 | |
| 117 | 395.1495 | |
| 118 | 559.4174 | |
| 119 | 749.538 | |
| 120 | 661.4857 | |
| 121 | 383.3144 | |
| 122 | 705.5124 | |
| 123 | 617.4598 | |
| 124 | 691.4958 | |
| 125 | 511.2095 | |
| 126 | 603.4434 | |
| 127 | 573.434 | |
| 128 | 647.4696 | |

| | | |
|---|---|---|
| 129 | 547.2807 | |
| 130 | 529.4073 | |
| 131 | 837.5908 | |
| 132 | 441.3602 | |
| 133 | 366.3731 | |
| 134 | 659.2876 | |
| 135 | 468.4413 | |
| 136 | 282.2794 | Spectral Match to 9-Octadecenamide; (Z)- from NIST14 |
| 137 | 679.3638 | |
| 138 | 440.41 | |
| 139 | 643.2929 | |
| 140 | 1273.604 | |
| 141 | 205.1584 | |
| 142 | 965.2498 | |
| 143 | 425.3776 | |
| 144 | 798.5213 | |
| 145 | 871.5749 | Pheophytin A |
| 146 | 369.0947 | |
| 147 | 731.1778 | |
| 148 | 693.2773 | |
| 149 | 489.4031 | |
| 150 | 811.4985 | |
| 151 | 339.3459 | |
| 152 | 408.2138 | |
| 153 | 638.3387 | |
| 154 | 487.1622 | |
| 155 | 387.1851 | |
| 156 | 309.2062 | |
| 157 | 540.4262 | |
| 158 | 111.0153 | |
| 159 | 811.3095 | |
| 160 | 675.4059 | |
| 161 | 777.5495 | |

| | | |
|---|---|---|
| 162 | 485.1422 | |
| 163 | 621.3105 | |
| 164 | 572.181 | |
| 165 | 683.4639 | |
| 166 | 960.5736 | |
| 167 | 725.6167 | |
| 168 | 711.4959 | |
| 169 | 959.5699 | |
| 170 | 376.3185 | |
| 171 | 1572.048 | |
| 172 | 609.2711 | |
| 173 | 763.5184 | |
| 174 | 413.1573 | Dihydro-lactucopicrin |
| 175 | 1263.5958 | |
| 176 | 1151.4238 | |
| 177 | 1023.6294 | |
| 178 | 489.2275 | |
| 179 | 679.3639 | |
| 180 | 795.3572 | |
| 181 | 390.3128 | |
| 182 | 790.3799 | |
| 183 | 845.4136 | |
| 184 | 951.3348 | |
| 185 | 1325.9017 | |
| 186 | 1290.6303 | |
| 187 | 1683.2051 | |
| 188 | 719.5256 | |
| 189 | 625.2664 | |
| 190 | 609.2711 | |
| 191 | 695.3591 | |
| 192 | 661.3031 | |
| 193 | 763.5525 | |
| 194 | 1746.2046 | |

| | |
|---|---|
| 195 | 1688.1628 |
| 196 | 1916.3717 |

**Suppelmentary File 3**

| Name | Parent mass | Compound_Name |
|---|---|---|
| 1 | 261.112 | 8-Deoxy-lactucin |
| 2 | 411.143 | Lactucopicrin |
| 3 | 277.215 | Spectral Match to 9S-Hydroxy-10E;12Z;15Z-octadecatrienoic acid from NIST14 |
| 4 | 338.341 | Spectral Match to 13-Docosenamide; (Z)- from NIST14 |
| 5 | 277.106 | Lactucin |
| 6 | 346.258 | |
| 7 | 609.269 | |
| 8 | 279.231 | |
| 9 | 197.117 | |
| 10 | 543.197 | |
| 11 | 364.32 | |
| 12 | 293.21 | |
| 13 | 377.265 | |
| 14 | 263.127 | Dihydro-8-deoxy-lactucin |
| 15 | 609.269 | |
| 16 | 521.216 | |
| 17 | 458.31 | |
| 18 | 259.096 | |
| 19 | 646.501 | |
| 20 | 443.169 | |
| 21 | 291.231 | |
| 22 | 296.258 | |
| 23 | 283.093 | |
| 24 | 540.388 | |
| 25 | 342.335 | |
| 26 | 181.122 | |
| 27 | 593.274 | |
| 28 | 611.465 | |
| 29 | 279.231 | |
| 30 | 315.192 | |
| 31 | 293.21 | |

| | | |
|---|---|---|
| 32 | 423.273 | |
| 33 | 317.207 | |
| 34 | 348.272 | |
| 35 | 279.122 | 11-B;13-Dihydro-lactucin |
| 36 | 532.347 | NCGC00380867-01_C27H46O9_9;12;15-Octadecatrienoic acid; 3-(hexopyranosyloxy)-2-hydroxypropyl ester; (9Z;12Z;15Z)- |
| 37 | 307.225 | |
| 38 | 442.206 | |
| 39 | 465.102 | Spectral Match to Isoquercitin from NIST14 |
| 40 | 291.194 | |
| 41 | 275.2 | Spectral Match to 9-OxoOTrE from NIST14 |
| 42 | 395.148 | |
| 43 | 351.213 | |
| 44 | 375.25 | |
| 45 | 263.127 | |
| 46 | 479.081 | |
| 47 | 328.247 | |
| 48 | 295.226 | 13-OxoODE |
| 49 | 449.107 | Spectral Match to Astragalin from NIST14 |
| 50 | 613.481 | |
| 51 | 259.205 | |
| 52 | 667.452 | |
| 53 | 394.243 | |
| 54 | 214.143 | |
| 55 | 773.517 | |
| 56 | 662.497 | |
| 57 | 808.555 | |
| 58 | 181.122 | |
| 59 | 309.205 | |
| 60 | 554.346 | |
| 61 | 294.133 | |
| 62 | 351.252 | |
| 63 | 685.462 | |
| 64 | 353.267 | Spectral Match to Monolinolenin (9c;12c;15c) from NIST14 |

| | | |
|---|---|---|
| 65 | 694.398 | |
| 66 | 452.32 | |
| 67 | 179.033 | |
| 68 | 370.294 | |
| 69 | 667.449 | |
| 70 | 305.246 | Spectral Match to 15-OxoEDE from NIST14 |
| 71 | 235.168 | |
| 72 | 275.2 | |
| 73 | 553.424 | |
| 74 | 425.179 | NCGC00385202-01_C21H28O9_ |
| 75 | 333.203 | |
| 76 | 406.242 | |
| 77 | 537.319 | |
| 78 | 281.172 | |
| 79 | 341.086 | Massbank:PR100475 Esculin\|Esculoside\|6;7-Dihydroxycoumarin-6-glucoside\|Aesculin\|Esculetin-6-beta-D-glucopyranoside\|Aesculinum |
| 80 | 425.213 | |
| 81 | 394.351 | |
| 82 | 391.244 | |
| 83 | 623.284 | |
| 84 | 438.378 | |
| 85 | 280.174 | NCGC00380275-01_C12H22O6_beta-D-Glucopyranoside; (3Z)-3-hexen-1-yl |
| 86 | 397.33 | |
| 87 | 611.465 | |
| 88 | 654.456 | |
| 89 | 639.279 | |
| 90 | 425.373 | |
| 91 | 371.206 | |
| 92 | 408.367 | |
| 93 | 596.309 | |
| 94 | 312.252 | |
| 95 | 409.272 | |
| 96 | 387.2 | roseoside |
| 97 | 393.28 | |

| | |
|---|---|
| 98 | 404.227 |
| 99 | 628.193 |
| 100 | 702.211 |
| 101 | 482.404 |
| 102 | 618.325 |
| 103 | 179.106 |
| 104 | 601.299 |
| 105 | 360.273 |
| 106 | 429.152 |
| 107 | 317.208 |
| 108 | 293.21 |
| 109 | 331.223 |
| 110 | 667.449 |
| 111 | 538.351 |
| 112 | 310.31 |
| 113 | 276.216 |
| 114 | 575.185 |
| 115 | 326.268 |
| 116 | 611.426 |
| 117 | 207.137 |
| 118 | 403.231 |
| 119 | 275.2 |
| 120 | 607.289 |
| 121 | 609.446 |
